# Frequency of Interferon-Resistance Conferring Substitutions in Amino Acid Positions 70 and 91 of Core Protein of the Russian HCV 1b Isolates Analyzed in the T-Cell Epitopic Context

**DOI:** 10.1155/2018/7685371

**Published:** 2018-02-07

**Authors:** V. S. Kichatova, K. K. Kyuregyan, N. V. Soboleva, A. A. Karlsen, O. V. Isaeva, M. G. Isaguliants, M. I. Mikhailov

**Affiliations:** ^1^Chumakov Federal Scientific Center for Research and Development of Immune-and-Biological Products of Russian Academy of Sciences, Moscow 108819, Russia; ^2^Russian Medical Academy of Continuous Professional Education, Moscow 125993, Russia; ^3^Mechnikov Research Institute for Vaccines and Sera, Moscow 105064, Russia; ^4^NF Gamaleja Research Center of Epidemiology and Microbiology, Moscow 123098, Russia; ^5^Riga Stradins University, Riga LV-1007, Latvia

## Abstract

Amino acid substitutions R70Q/H and L91M in HCV subtype 1b core protein can affect the response to interferon and are associated with the development of hepatocellular carcinoma. We found that the rate of R70Q/H in HCV 1b from Russia was 31.2%, similar to that in HCV strains from Asia (34.0%), higher than that in the European (18.0%, *p* = 0.0010), but lower than that in the US HCV 1b strains (62.8%, *p* < 0.0001). Substitution L91M was found in 80.4% of the Russian HCV 1b isolates, higher than in Asian isolates (43.8%, *p* < 0.0001). Thus, a significant proportion of Russian HCV 1b isolates carry the unfavorable R70Q/H and/or L91M substitution. In silico analysis of the epitopic structure of the regions of substitutions revealed that both harbor clusters of T-cell epitopes. Peptides encompassing these regions were predicted to bind to a panel of HLA class I molecules, with substitutions impairing peptide recognition by HLA I molecules of the alleles prevalent in Russia. This indicates that HCV 1b with R70Q/H and L91M substitutions may have evolved as the immune escape variants. Impairment of T-cell recognition may play a part in the negative effect of these substitutions on the response to IFN treatment.

## 1. Introduction

Approximately 177.5 million people worldwide are infected with the hepatitis C virus (HCV). In 60–80% of cases, infection results in the chronic liver disease; 10 to 25% progress to cirrhosis and hepatocellular carcinoma (HCC) [1–4], the second most common cause of cancer death worldwide after the lung cancer [5]. HCV infection is associated with at least half of the HCC cases [6]. In patients with hepatitis C, treatment with the direct-acting antivirals (DAAs) of the second generation results in the sustained virological response (SVR) in 98-99% cases. Unfortunately, due to the high cost and limited availability of DAAs, clinical practice in low- to moderate-income countries still relies on the pegylated interferon and ribavirin (PEG-IFN/RBV) therapy.

Infections with HCV genotype 1 that still predominate (in 48% of HCV-infected people), specifically HCV 1b (in 23% of HCV-infected people [4]), have been repeatedly associated with poor prognosis [7–11]. In chronic hepatitis C, infection with HCV 1b increases the rate of progression to fibrosis and cirrhosis and the risk of HCC development compared to infections with other HCV genotypes [8–12]. HCV genotype 1 infection is also an important prognostic factor of poor SVR to PEG-IFN/RBV treatment, with the response rate for genotypes 2 and 3 reaching 80%, and for genotype 1, only 35 to 45% [13–15], being lower or tending to be lower for HCV 1b carriers (37% of patients with subtype 1b and 45% of those with subtype 1a [16]). Several studies and meta-analyses have concluded that eradication of HCV with antiviral therapy reduces the risk of HCC in patients with chronic hepatitis C, but the risk is not eliminated [17]. Wide spread of HCV 1b, high rate of progression to chronic hepatitis C, poor response to PEG-IFN/RBV therapy still actual in poor to modest healthcare settings, and the increased risks of HCC development prompt continuation of the studies on the mechanisms of HCV 1b resistance to interferon treatment.

Akuta et al. were the first to report that amino acid substitutions in positions 70 and 91 of the HCV 1b core protein associate with virological response to PEG-IFN/RBV treatment. They found that change of arginine to glutamine or histidine in amino acid (aa) position 70 (R70Q and R70H or R70Q/H) and/or methionine in aa position 91 (L91M) was present in 100% of nonresponders who tested positive for HCV RNA at the end of 48 weeks of PEG-IFN/RBV treatment, but in only 42% of responders [18]. The significance of R70Q for HCV 1b (and also for subtype 5a) was confirmed in a series of later studies (see [19] for a review). R70Q was recorded as a predictive marker for the resistance to PEG-IFN/RBV also in the prolonged treatment regimens (72 weeks) and in the triple therapy with PEG-IFN/RBV and second-generation DAA [19–21]. A series of studies determined the frequency of these mutations in different population groups [22–24]. All, including the very recent ones, concluded that the nature of amino acid residues in aa position 70 of HCV core can help to distinguish patients who can still benefit from the affordable IFN-based therapy from those who must be treated with DAAs to prevent the evolution towards the end-stage liver disease [25]. The clinical significance of substitutions in aa position 91 of HCV 1b is less well defined [19] and for other HCV genotypes either unclear [19] or not demonstrated [26–29]. Interestingly, clinical and experimental studies demonstrated the involvement of both of these mutations in hepatocellular carcinogenesis [30, 31]. Amino acid substitution R70Q/H appeared to be associated with cirrhosis and development of HCC even in PEG-IFN/RBV-treated patients achieving SVR [17, 22, 32–38]. Altogether, this motivates the need to characterize polymorphisms in HCV 1b core protein and identify patients carrying “carcinogenic” HCV 1b variants staying at a high risk of developing HCC even after the successful treatment.

The prevalence of HCV core polymorphisms R70Q/H and/or L91M in the territory of the Russian Federation was not characterized despite high incidence of HCV infections [39], the predominance of HCV 1 genotype, specifically of HCV 1b subtype (up to 50% [40–42]), and wide use of standard interferon therapy. In this study, we filled this gap and described the spread in the Russian Federation of HCV 1b strains with R70Q/H and/or L91M substitutions in comparison to their spread in the other geographical regions and evaluated the effect of these substitutions on the recognition of these regions by the immune system of the patients.

## 2. Materials and Methods

### 2.1. Samples

The analysis included 313 sequences encoding HCV core amplified from the serum samples of patients with chronic hepatitis C collected during 2007–2014 in different regions of the Russian Federation: Moscow and the Moscow region (*n* = 238), Rostov/Rostov-on-Don (*n* = 12), Tuva/Kyzyl (*n* = 12), Khabarovsk (*n* = 36), Sverdlovsk/Yekaterinburg (*n* = 4), and Sakha/Yakutsk (*n* = 11). Age and gender were known for 117 individuals; 34 were females and 83 were males (the female to male ratio was 1 : 2.4). Mean age was 40 ± 14 years. All serum samples included into the database were from the general population with the exception of those from Moscow/Moscow region represented by the subgroup of the general population without known risk factors (*n* = 49) and intravenous drug users (IDUs; *n* = 45). Written informed consent was obtained from each patient.

### 2.2. HCV Genotyping and Sequencing

HCV genotype and subtype were determined by the phylogenetic analysis of HCV core coding region; and for HCV 2k/1b recombinants, also of the NS5b region. Nucleic acid extraction was performed using QIAamp Viral RNA Mini Kit (QIAGEN, Hilden, Germany), or MagNA Pure Compact Nucleic Acid Isolation Kit I (Roche Applied Science, Mannheim, Germany), or Sileks MagNA kit (Sileks, Moscow, Russia). Reverse transcription and amplification was performed with Transcriptor First Strand cDNA Synthesis Kit and Fast Start High Fidelity PCR System (Roche Applied Science). HCV core coding region (nucleotide positions 273–1315 according to HCV 1a reference strain H77, GenBank accession number AF011753) was subjected to nested PCR with the following primers: outer forward 5′-GCT AGC CGA GTA GTG TTG GG-3′; outer reverse 5′-ACC AGT TCA TCATCA TAT YCC-3′; inner forward 5′-GAA AGG CCT TGT GGT ACT GC-3′; and inner reverse 5′-TTC ATC ATA TTC CAT GCCA-3′. The first and the second steps of PCR were as follows: 5 min at 94°C, then 35 cycles of 45 sec at 94°C, 45 sec at 55°C, 90 sec at 72°C, and final elongation for 7 min at 72°C. PCR products of 1043 nt length were cut and extracted from agarose gel using QIAquick Gel Extraction kit (QIAGEN). Sequencing was performed on an automated sequencer 3130 Genetic Analyzer (ABI, Foster City, USA) using Big Dye Terminator v.3.1 Cycle Sequencing Kit according to the manufacturer's protocol. The genotype was established by comparison sequences with GenBank reference sequences representative for all HCV genotypes.

### 2.3. HCV Sequence Database

Database of 313 HCV sequences was supplemented with 94 sequences from GenBank geographically attributed to St. Petersburg (*n* = 42: AY070167–AY070169, AY070174.1, AY070178–AY070211, AY070214, AY070215, AY587844, and AY587845); Novosibirsk (*n* = 47: DQ001223–DQ001264, DQ001267–DQ001270, AH014196.2, and AH014197.2); and Moscow (*n* = 5: AF176573, KM054515, KM054516, KT983617, and X71407). The complete database consisted of 407 sequences of the “Russian” HCV core of subtypes: 1a (*n* = 29), 1b (*n* = 189), 3a (*n* = 171), and recombinant 2k/1b (*n* = 18) (Table 1). Group of comparison consisted of 2210 HCV core sequences from the Los Alamos database (LANL; https://hcv.lanl.gov/content/index) distributed as follows: Asia (*n* = 374), USA (*n* = 1343), and Europe (*n* = 497) (Table 1). Samples of the Russian patients infected with HCV1b formed a separate set divided into the subsets depending on the region of sample collection (Table 2). The geographical distribution of these subsets is illustrated by Supplementary Figure S1.

### 2.4. In Silico Analysis of HCV Core Sequences

Alignment of the nucleotide and predicted amino acid sequences of HCV were performed using MEGA 7.0.18. Prevalence of substitutions in amino acid positions 70 and 91 was calculated using Microsoft Office Excel. Phylogenetic analysis of HCV1b core sequences (Supplementary Figure S2) was done using a Bayesian likelihood-based algorithm implemented in Beast version 1.8.4 [43]. The SRD06 substitution model was used with a relaxed lognormal clock. Analysis was run over 700 million generations and trees were sampled every 70,000 generations, resulting in 70,000 trees. Trees were annotated with TreeAnnotator v.1.8.3 using a burn-in of 1000 trees and visualized with FigTree v.1.4.2 (Andrew Rambaut; http://tree.bio.ed.ac.uk/software/figtree/). Search for T-cell epitopes in HCV core sequences was performed through EIDB database (http://www.iedb.org) [44]. Epitope sequences were aligned with HCV core aa sequences using MEGA software.

### 2.5. Prediction of Presentation of HCV Core-Derived Peptides by HLA Class I and II Molecules

Amino acid sequences of HCV core of 189 HCV 1b were used to build the consensus sequence of “Russian” HCV 1b core (sequence in GenBank deposition). Consensus sequence was divided into nine amino acid- (aa-) long peptides with mandatory presence of aa residues 70 or 91. Prediction of presentation of resulting HCV core-derived peptides by human leukocyte antigen class I molecules (HLA class I) was done on EPITOPE VACCINE OPTIMIZATION server (http://bio.med.ucm.es/episopt.html) using EPISPOT tool (http://bio.med.ucm.es/episopt.html; [45]). Analysis was carried for a selection of HLA I alleles prevalent in the Russian population based on the most representative dataset [46] and other data deposited in the Allele Frequency Net Database (http://www.allelefrequencies.net/pop6001c.asp?pop_id=3322; [47]).

Presentation by HLA class II molecules was evaluated using the NetMHCIIpan 3.1 program (http://www.cbs.dtu.dk/services/NetMHCIIpan-3.1) [48–50] for the region starting with 20-mer peptide with the C-terminal aa 70 and ending with the 20-mer peptide with the N-terminal aa 91. Analysis was done for the following: 275 HLA class II alleles DRB1^∗^1101–DRB1^∗^1195, DRB1^∗^1301–DRB1^∗^1399, and DRB1^∗^1501–DRB1^∗^1549; and combination of alpha and beta chains DQA1^∗^0101, DQA1^∗^0102, DQA1^∗^0301, DQA1^∗^0501 with DQB1^∗^0201, DQB1^∗^0301, DQB1^∗^0501, DQB1^∗^0602, DQB1^∗^0603, DQB1^∗^0604, DQB1^∗^0605, DQB1^∗^0606, DQB1^∗^0607, and DQB1^∗^0608, selected based on the published data on the frequency of HLA class II alleles in the Russian Federation [46, 47, 51]. Strong binding was characterized by binding level < 2%; weak binding, 2 to 10%; no binding, >10%.

### 2.6. Statistical Analysis

Data analysis was performed using the http://Graphpad.com/. Statistical significance was evaluated by Fisher's exact test using parametric model, two-tailed *p* value < 0.05 was considered statistically significant.

## 3. Results

### 3.1. Characteristics of the Set of HCV 1b Sequences Used in the Study

The analysis included 313 sequences encoding HCV core amplified from the serum samples of patients with chronic hepatitis C collected during 2007–2014 from the general population in different regions of the Russian Federation, supplemented with 94 sequences from the GenBank (*n* = 407; Table 1). Sequence set included 189 HCV 1b sequences derived from the territory of the Russian Federation, 140 obtained here, and 49 sequences from the GenBank. We performed their phylodynamic analysis with the focus on 140 HCV 1b isolates (Supplementary Figure S2). The analysis illustrated a long history of HCV 1b evolution on the Russian territory, with current strains having an average time of separation from the “foreign” strains of approximately 15 ± 5 years, the oldest strains separating 60 ± 15 years ago (Supplementary Figure S2). No clusters were revealed, indicating that sequences were not related. HCV 1b sequences with R70Q/H and/or L91M substitutions did not form any clusters either, being distributed evenly throughout the phylogenetic tree (Supplementary Figure S2). This demonstrates that the selected sequences are geographically genuine, adequately reproduce the diversity of HCV 1b in the territory of the Russian Federation, and are, therefore, suitable for the analysis of frequency of occurrence of amino acid substitutions in positions 70 and 91 in the territory of the Russian Federation.

### 3.2. Frequency of Substitutions in aa Positions 70 and 91 of HCV 1b from the Russian Federation Compared to Other Geographical Regions

Substitution R70Q was found in 27.0% and R70H in 4.2% of the Russian HCV 1b isolates, which was similar to their frequencies in the isolates from Asia and in all countries taken together (*p* > 0.05), but significantly higher than the frequency of R70Q and of R70H in Europe (17.7%, *p* = 0.0179; and 0.3%, *p* = 0.0021, resp.; Table 1). Substitution L91M was found in 80.4% of HCV 1b isolates from Russia, likewise to Europe and worldwide, but more often than in the strains from Asia (43.8%, *p* < 0.0001; Table 1). Substitution R70Q was significantly more prevalent compared to substitution R70H in all geographical regions including Russia. Frequency of occurrence of substitutions in positions 70 and 91 in HCV genotypes 1a and 3a was low (Table 1). Geographical distribution of amino acid variants in positions 70 and 91 for HCV subtypes 1a and 3a was similar, except for comparatively more frequent occurrence of R70Q in HCV 3a strains from Asia (significantly more than in the Russian HCV subtype 3a strains; 9.8% versus 2.9%, *p* = 0.0204; Table 1). All available core sequences of the recombinant HCV 2k/1b (18 from Russia and 4 from Europe) had no substitutions in either position 70 or 91 (data not shown).

### 3.3. Pattern of Substitutions in aa Positions 70 and 91 in HCV 1b Strains in the Russian Federation

**Figure 1:**
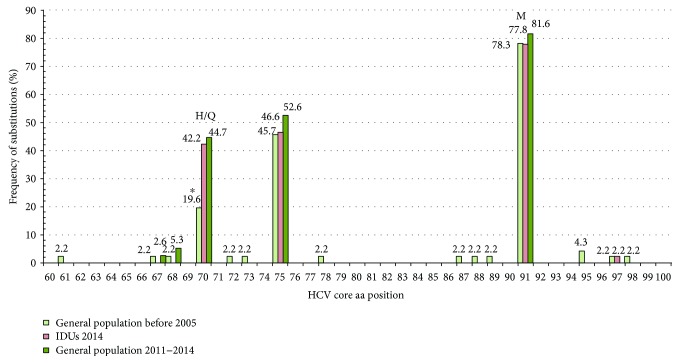
Frequency of amino acid substitutions in the region between aa 60 to 100 of the nucleocapsid (core) of HCV 1b, in samples collected before 2005 in the general population in Russia (“general population before 2005”; *n* = 46), during 2011–2014 in Moscow/Moscow region in the general population (“general population 2011–2014”; *n* = 38), and in intravenous drug users (“IDUs 2014”; *n* = 45). ^∗^Frequency of occurrence of R70Q/H in the group “general population before 2005” (19.6%) was significantly lower compared to “general population 2011–2014” (44.7%, *p* = 0.0178) and “IDUs 2014” (42.2%, *p* = 0.0240), while frequency of the substitution in the latter two groups did not differ.

HCV 1b variants with substitutions R70Q/H and L91M were evenly distributed in all eight regions of the Russian Federation assessed (Table 2). Differences were observed only between Moscow (R70Q/H, 40.4%; L91M, 79.8%) and Novosibirsk subcohorts (R70Q/H, 20.7%, *p* = 0.0049; L91M, 75.5%, *p* = 0.0094). Substitution R70Q dominated over substitution R70H in all studied cohorts (data not shown). Prevalence of R70Q/H and L91M mutations were similar in males and females (*р* = 0.8286 and *p* = 0.439 for aa positions 70 and 91, resp.). Comparison of subcohorts with a known year of sample collection demonstrated the accumulation of R70Q/H substitution over time: they were found in 19.6% of samples collected before 2005 and in 39.1% in samples collected in 2011–2014 (*p* = 0.038; Table 2). An increase in the frequency of occurrence of the substitution was observed only for position 70 but not for 91 (Figure 1).

Analysis of the Moscow cohort demonstrated similar frequency of R70Q/H and L91M mutations among the general population and the intravenous drug users (IDUs) (R70Q/H: 38.7% versus 42.2%, *p* = 0.8341; L91M: 81.6% versus 77.7%, *p* = 0.7979; Table 2). Difference between these two population groups was observed only in aa residue in position 75: R75H/Q occurred in the general population more frequently than in IDUs (52.6% and 46.6%, resp.; *p* = 0.0423). Substitutions at aa position 75 were repeatedly detected in the earlier studies [52, 53], but their clinical significance is yet unclear.

### 3.4. Epitopic Analysis of the Region of HCV Core Containing Amino Acid Residues 70 and 91

Amino acid sequences of HCV core of 189 HCV 1b were used to build the consensus sequence of the “Russian” HCV 1b core, an “average” HCV sequence largely free from the patient-specific adaptations [54]. Epitopic analysis of the consensus HCV core using Immune Epitope Database and Analysis resource (http://www.iedb.org) identified 183 CD4+ and CD8+ Т cell epitopes, with 14 (7.7%) encompassing aa 70 and 29 (15.8%) encompassing aa 91, respectively. Totally, 23.5% of all T-cell epitopes within HCV core included either aa position 70 or 91. HCV 1b core was found to harbor 8 epitopes encompassing aa 70 and 11 encompassing aa 91 (Supplementary Figure S3).

### 3.5. Prediction of Recognition of the Region of HCV 1b Core Containing Amino Acid Residues 70 and 91 by HLA Class I and II Molecules

**Figure 2:**
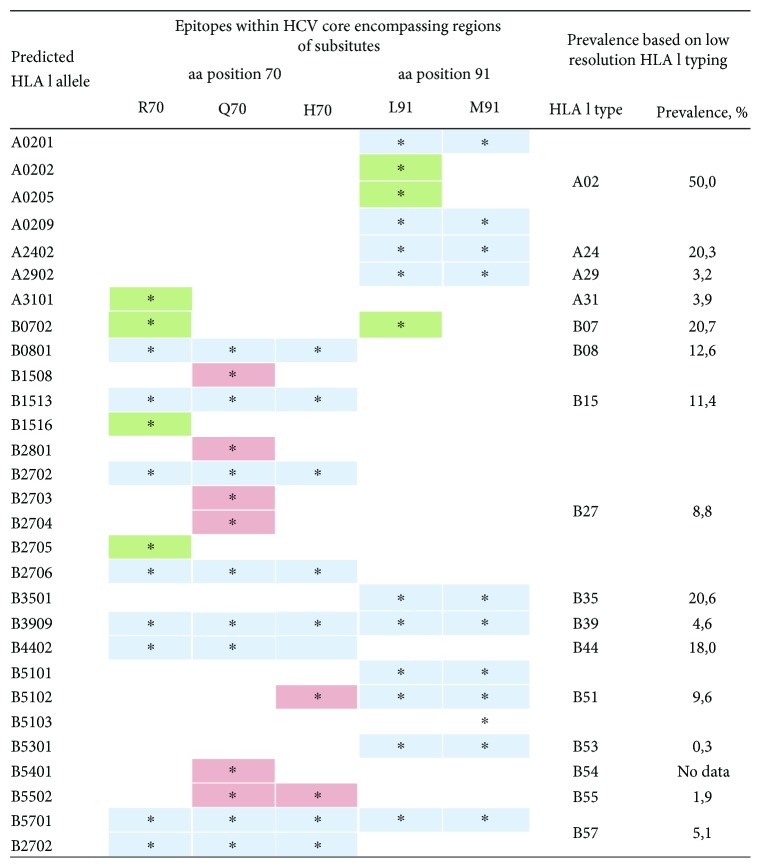
Prediction of binding of peptides encompassing aa 70 and 91 of nucleocapsid (core) protein of HCV 1b to HLA I alleles prevalent in the Russian population. Binding prediction was done using EPISPOT tool (http://bio.med.ucm.es/episopt.html [45]). Allele frequency data is based on the data obtained from Allele Frequency Net Database [46, 47]. Predicted binding is depicted by an asterisk. HLA class I molecules binding only to the peptides containing wild-type amino acid residues R70 and/or L91 are shaded green; only variants with Q70, or H70, and/or M91 mutations, red; binding regardless of the nature of amino acid residues in positions 70 and/or 91, blue.

First, we analyzed how HCV core variants with and without mutations were recognized by HLA class I molecules. The complete list of HLA I alleles and peptides within HCV 1b core capable to bind to them is presented in Supplementary Table S1 for regions aa 62 to 78 and S2 for aa 83 to 99. From these lists, we selected HLA I alleles prevalent in the territory of the Russian Federation [28, 29] (Figure 2). Several alleles predicted to bind peptides with the wild type, but not with the mutant amino acid residues, were identified. For peptides encompassing aa position 70, these were A3101, B0702, and B1516 and for aa position 91, A0202, A0205, and B0702 (Figure 2, Tables S1 and S2). HLA-B0702 allele, prevalent in the Russian population (20.7%), turned to be of particular interest as it was predicted to bind peptides containing R70 and L91 but not their mutated variants containing Q70, or H70, and M91 (Figure 2, Tables S1 and S2).

We have also assessed if HCV core variants with and without substitutions can bind to HLA class II molecules. Since HLA class II are capable of binding up to 20 amino acid long peptides, we included in the analysis the consensus amino acid sequence of HCV 1b spanning 39 aa with aa positions 70 or 91 in the middle. Data on the frequency of HLA II alleles in the Russian population with high-resolution typing are limited. Based on the low-resolution analysis for HLA II DR, the most prevalent alleles are HLA II DRB101 (22.4%), DRB104 (20.5%), DRB107 (25.6%), DRB111 (26.7%), DRB113 (26.0%), and DRB115 (23.4%) [28, 29]. High-resolution data on the frequency of HLA II DQA1 and DQB1 alleles for ethnical Russians is available for only one geographical region of the Russian Federation (Astrakhan region) [30]. According to this data, the most prevalent alleles are as follows: DQA1^∗^0101 (24.0%), DQA1^∗^0102 (34.3%), DQA1^∗^0301 (20.0%), DQA1^∗^0501 (47.3%), DQB1^∗^0201 (33.7%), DQB1^∗^0301 (39.3%), DQB1^∗^0501 (20.3%), and DQB1^∗^0602-08 (36.0%) [30]. Analysis of binding of these HLA II molecules with HCV core-derived peptides done using NetMHCIIpan program included the total of 275 alleles. None of the HLA II DRB1 alleles prevalent in Russia were predicted to bind any aa fragment within the consensus HCV 1b core sequence spanning aa positions 50 to 110. Weak binding with the fragment aa 71 to 110 harboring aa 91 was predicted for alleles HLA-DQA1^∗^0501-DQB1^∗^0301, HLA-DQA1^∗^0501-DQB1^∗^0501, HLA-DQA1^∗^0501-DQB1^∗^0603, HLA-DQA1^∗^0501-DQB1^∗^0604, HLA-DQA1^∗^0501-DQB1^∗^0607, HLA-DQA1^∗^0501-DQB1^∗^0608, HLA-DQA1^∗^0301-DQB1^∗^0301, and HLA-DQA1^∗^0301-DQB1^∗^0501 (exemplified by the data set in the Supplementary Table S3). The binding was not affected by the nature of amino acid residue in position 91. No binding was predicted for the peptides spanning aa 50 to 90 harboring aa 70 (data not shown).

## 4. Discussion

The presence of substitutions R to Q/H in aa position 70 of the nucleocapsid (core) of HCV 1b can help distinguish patients who can still benefit from the affordable IFN-based therapy from those who do not respond and are at an increased risk of HCC development [17, 22, 25, 32–38]. There are also indications of the clinical significance of L to M substitution in aa position 91 of HCV 1b, although less well defined [18, 19]. Here, we for the first time analyzed the frequency of occurrence of R70Q/H and L91M substitutions in a set of 189 sequences of HCV 1b isolates derived from the territory of the Russian Federation. Phylogenetic analysis has shown that these sequences formed no clusters, and it evolved as a result of continuous viral circulation in the territory of the former Soviet Union, which indicates that they adequately represent the diversity of Russian HCV 1b isolates. We found that 31.4% of the Russian HCV 1b isolates harbor R70Q/H, that is, almost one-third of individuals infected with HCV 1b in the Russian Federation carry viral isolates that are potentially resistant to the standard PEG-IFN/RBV therapy and confer an increased risk of HCC development. Comparative analysis of HCV 1b core variant distribution demonstrated a higher prevalence of unfavorable R70Q/H variants in the Russian Federation compared to the neighboring European countries. USA was the only region where the frequency of R70Q mutation appeared to be higher. At the same time, L91M substitution was found to be present in the majority of HCV 1b strains circulating in all regions of the world, including the Russian Federation (except for Asia, where the predominant viral variant had L91). Analysis of the frequency of R70Q/H and L91M in HCV 1b isolated in the different regions of the Russian Federation demonstrated the ubiquitous even distribution of these variants. Example of Moscow/Moscow region has also shown that R70Q/H and L91M HCV 1b variants had similar prevalence in the general population and in the high-risk group of intravenous drug users from the same geographical region, which excluded the influence of this risk group on the results of this study.

We have analyzed the prevalence of substitutions R70Q/H and L91M in all HCV 1b core sequences from LANL (*n* = 898) regardless of the area of sample collection. Similar analysis has been performed by two independent research groups in 2010 [26] and in 2014 [19]. The frequency of occurrence of R70Q and R70H substitutions in HCV 1b isolates in the study done in 2010 was significantly higher than in our analysis (60% and 4% versus 32.9% and 1.7%, resp., for all samples regardless of the time of collection; *p* < 0.01), whereas the frequency of occurrence of L91M substitution was similar (71% in 2010 [26] versus 69% in 2016, *p* > 0.05). Interestingly, we registered an increase in the frequency of occurrence of R70Q/H variant in the Russian Federation between 2005 and 2014: R70Q/H variant was present in 19.6% of samples collected before 2005 and in 39.1% in samples collected in 2011–2014 (*p* < 0.05; Table 2). This pointed at a positive selection of this variant in the Russian population with its prevalence increasing towards the levels observed worldwide.

We observed an increase in the frequency of substitutions in aa position 70, not elsewhere in this region of HCV core, suggesting that this substitution was not related to the changes in the other regions of the core protein. The latter is supported by the absence of covariance of the residues in amino acid position 70 with amino acid residues in other positions of HCV core, or elsewhere in HCV polyprotein [55]. This, together with the long history of evolution of HCV 1b strains in the Russian territory, suggests that substitutions in aa position 70 of HCV 1b core protein are not the compensatory ones. Although, Tasaka-Fujita et al. found polymorphisms at aa 70 to be associated with the efficiency of *in vitro* production of the infectious virus, with deteriorated virus production in 70Q strains resulting in the intracellular accumulation of HCV proteins [30]. Also, a recent study done in 112 Chinese patients with chronic HCV 1b infection revealed that in patients infected with mixtures of 70R and 70Q/H HCV 1b strains (most of the patients in this study), viral kinetics of two strains changed synchronously during the treatment [56]. The latter data indicated that R70Q/H substitutions (i) do not improve viral replication fitness; (ii) have not evolved as a result of the selective pressure of PEG-IFN*α*/RBV treatment; and (iii) do not confer an advantage in the viral replication/propagation under the treatment conditions [56]. Altogether, these point that positive selection of R70Q/H is unrelated to the viral replication fitness, stressing the importance in the selection of the host-related factors.

Host factors are multiple and include alcohol consumption, age at infection, infections/coinfections, and genetic factors, such as gender, or single-nucleotide polymorphisms upstream of IL-28B [57–60]. Funaoka et al. using a virus culture system demonstrated the suppression of IFN signaling (IFN resistance) in cultured cells infected with 70Q/H HCV 1b strains HCV 1b infected with core mutation (70Q/H), possibly induced by the IL-6-induced upregulation of SOCS3 [61]. HCV 1b-infected patients with 70Q had significantly higher homeostasis model assessment insulin resistance (HOMA-IR) scores compared to HCV 1b patients without substitution in this position, suggesting that the substitution has a close relationship to insulin resistance [62]. Mechanistically, extracellular HCV core protein with substitution at position 70 was found to enhance IL-6 production and reduce adiponectin production from visceral adipose tissue, which can cause insulin resistance, hepatic steatosis, and ultimately development of HCC [63]. This, as well as other experimental and clinical studies identified a series of associations between substitutions in HCV core and such host-related factors as oxidative stress and HCV-induced metabolic disorders [64–66].

An important host genetic factor appeared to be the ethnicity. We have looked for the published data on the links between the response to standard treatment, host ethnicity, and prevalence of core mutations in different population groups. Unusually, poor response to PEG-IFN*α*/RBV treatment was repeatedly reported in the Afro-American patients compared to Caucasian Americans [67]. People of the African descent had lower chances of success with dual antiviral therapy compared with Caucasians. This was observed in the homogeneous populations with low rates of racial admixture assessed by self-reported ancestry, as well as in admixed populations when ancestry was assessed using genetic markers [68]. American patients of the African origins were found to less likely respond to treatment and achieve SVR than non-African-American patients [69]. Infected with genotype 1, they exhibited significantly lower decreases in the first-phase viral RNA, slower elimination of infected cells, and smaller declines in mean viral RNA over 1 month suggesting an impaired ability to block viral production in African-Americans [70]. On the contrary, Asian patients achieve higher sustained virologic response rates following IFN-based therapy than non-Asians [71]. Interestingly, for the Asian patients, superior virologic outcomes were observed also with different classes of DAAs alone or in combination [71] indicating that the outcomes were not determined by the effects of IFN. These observations fall in line with our findings that Asia is the place where the largest proportion of the virus has not yet acquired both R70Q/H and L91M mutations, whereas in America basically all HCV 1b variants are already mutated (occurrence of nonmutated sequences in America is significantly lower than in Asia, Russia, or Europe; *p* < 0.0005; Table 1).

Also, the treatment outcomes for chronic hepatitis C related to aa substitutions in aa positions 70 and 91 varied with ethnicity. The first studies demonstrating the importance of R70Q/H and L91M substitutions in the core protein were carried in patients infected with HCV 1b in Japan (18). However, they were not reproduced in all Japanese studies. Enomoto et al. identified the role in treatment response only of the amino acid substitutions in HCV NS5a, but not in the core protein [72]. A study of HCV 1b-infected Caucasian patients in Spain demonstrated a significant association with PEG-IFN/RBV treatment outcome with substitutions R62G, R70Q, and N110T [28]; in Saudi patients, with substitutions R70Q and A75; in Azerbaijani patients from Iran, with substitutions K43R, R70Q, L91M, and S106 [23]. Studies of the Caucasian patients infected with HCV 1b in Sweden, and in Caucasians infected with HCV 1b in the United States demonstrated significant association of PEG-IFN/RBV treatment outcome with substitutions in aa position 70, but not in other aa positions including 91 [26, 27]. Interestingly, despite the unusually poor response to PEG-IFN*α*/RBV treatment in the Afro-American patients compared to Caucasian Americans, their isolates did not differ in the frequency of occurrence of amino residues in position 70 [27]. Also, the association of differential viral responses with polymorphisms in core aa position 70 demonstrated in the North American patients was weaker than in the Japanese studies [27]. On top of it, in the study of the correlates of PEG-IFN*α*/RBV treatment response in the Chinese patients with chronic HCV 1b infection, 70Q/H HCV 1b strains exhibited the same virological response as the 70R strains [56]. Analysis of these studies demonstrated that the role of amino acid substitutions in positions other than 70 is discrepant, while substitutions in the position 70 predict poor IFN treatment response in patients of some, but not all ethnicities and nationalities.

While analyzing a broad scope of viral and host factors linking substitutions in aa positions 70 and 91 of HCV core with treatment response, these studies left out the issue of the adaptive immune response, and possible viral evolution under the pressure of the immune system of the host. Immune database analysis done here demonstrated that R70Q/H and L91M substitutions are localized in the T-cell epitopic clusters and may interfere with the immune recognition of host cells infected with mutant virions, which would affect the spontaneous and also IFN therapy-driven immune clearance. Immune recognition in populations of different ethnicity varies; different ethnic groups have distinct and characteristic HLA allele frequencies, resulting in the differential immune recognition of the epitope-carrying regions depending on the haplotype of the host, which would differentially drive viral immune escape. In this context, the increasing prevalence of mutant HCV variants can be explained by an escape from the dominant types of immune pressure in a certain population, in relation to the regionally prevalent HLA types. Such HLA allele-specific mutation patterns were earlier described for HIV-1 [73].

The assumption that HLA allele specific immune pressure would result in the regionally variable patterns of immune escape mutations falls in line with uneven geographical distribution of HCV 1b R70Q/H and L91M variants (Table 1). Furthermore, in silico analysis carried here suggests that at least some of HLA I alleles prevalent in the Russian Federation, such as A02 and B07, are able to bind 70R and 91L peptides but cannot bind peptides with R70Q/H or L91M substitutions. At the same time, the role of HLA class II binding, hence of the T-helper cell or antibody response in immune selection, was predicted as negligible. Due to insufficiency of the high-resolution HLA typing data for the Russian Federation, our predictions of HLA I and HLA II binding with HCV core peptides were done on the low-resolution level. Even with these limitations, our data points at the involvement of HLA I- (but not HLA II-) specific alleles in the differential immune recognition of HCV 1b variants with substitutions in aa positions 70 and 91, with the possibility for immune escape of the variants that have acquired R70Q/H and/or L91M. A very recent comparative analysis of the variation and selection in HCV genome demonstrated positive selection of HCV variants associated with HLA class I-driven CTL response (but not CD4+ T-cell response or RNA structure) [74]. Increasing frequency of occurrence of HCV 1b variants bearing R70Q/H and L91M may result from such positive selection. Interestingly, a study conducted in one particular region of the Russian Federation among patients of the Russian ethnicity found that HLA I haplotypes A02/B07 and A03/B07 are associated with the higher rates of spontaneous HCV elimination and protection against the development of chronic infection [51]. Carriage of A(^∗^)02 was also found to predict SVR in a study done in the Caucasian American patients [75]. In patients with chronic hepatitis C, HLA I allele B07 was related to the resistance to active chronic liver disease indicating its association with recognition and clearance of HCV-infected hepatocytes [51]. These findings support the clinical significance of our observations, requesting further confirmatory studies with high-resolution genotyping of the population.

## 5. Conclusions

This is the first study characterizing the frequency of occurrence of IFN resistance-conferring mutations in human hepatitis C virus isolates circulating in the territory of the Russian Federation, and the first one, in which the spread of viral variants with substitutions in aa positions 70 and 91 may be associated with the positive selection under the pressure of immune response. Spread of R70Q/H and L91M HCV 1b variants may result from an immune escape from the CTL response. The immunogenetic background of HCV-infected individuals (racial variations in viral-specific immunity) would then determine both the differences in geographical distribution of certain viral variants and ethnical differences in their response to treatment. The choice of treatment strategy for patients with HCV is increasingly based on the personalized approach. It takes into account many viral and host factors. Screening of HCV 1b-infected patients for unfavorable mutations in the core region could be a useful tool to identify the individuals in need of IFN-free treatment regimens employing DAA. In the future, after complete implementation of IFN-free regimens, analysis of polymorphisms at position 70 of HCV 1b core will remain relevant to identify patients at higher risk of HCC development who require immediate treatment.

## Figures and Tables

**Table 1:** Frequency of occurrence of substitutions at amino acid positions 70 and 91 of the nucleocapsid (core) protein of HCV genotypes 1a, 1b, and 3a in different population groups (in %).

HCV genotype	Substitutions	Geographical variation in % of sequences variants in HCV of different genotypes				
		Russia	Europe	Asia	USA	Total
1a	nn samples	*n* = 29	*n* = 149	*n* = 27	*n* = 1087	*n* = 1292
	Q70	3.4	0.7	0	1.7	1.5
	H70	0	0	0	0.1	0.1
	M91	0	0	0	0	0
	Both^∗^	0	0	0	0	0
	None^∗∗^	96.6	99.3	100	98.2	98.4
1b	nn samples	*n* = 189	*n* = 316	*n* = 224	*n* = 218	*n* = 947
	Q70	27.0	17.7	31.3	59.6	32.4
	H70	4.2	0.3	2.7	3.2	2.3
	M91	80.4	85.8	43.8	86.2	74.9
	Both^∗^	27.5	15.5	16.1	54.6	27.0
	None^∗∗^	15.9	11.7	38.4	5.5	17.4
3a	nn samples	*n* = 171	*n* = 28	*n* = 123	*n* = 38	*n* = 360
	Q70	2.9	7.1	9.8	0	5.3
	H70	0	0	0	0	0
	M91	0.6	0	0	0	0.3
	Both^∗^	0	0	0	0	0
	None^∗∗^	96.5	92.9	90.2	100	94.4

^∗^Substitutions in both aa 70 and 91; ^∗∗^no substitutions.

**Table 2:** Occurrence of substitutions in amino acid positions 70 and 91 of the nucleocapsid (core) protein of HCV 1b in the samples collected from the general population of the Russian Federation during the routine blood tests.

Number of cohort	Federal District of Russia	City	Date of collection of samples	Number of HCV 1b sequences	Mutations found in aa position 70, *n* (%)	Mutations found in aa position 91, *n* (%)	Mutations found in aa positions 70 and 91, *n* (%)	Without mutations in aa positions 70 and 91, *n* (%)
1	Central	Moscow	2008	5	1 (20.0)	4 (80.0)	1 (20.0)	1 (20.0)
			2009	6	1 (16.7)	5 (83.3)	0 (0)	0 (0)
			2011	17	10 (58.9)	15 (88.2)	9 (53.0)	1 (5.9)
			2014	21	7 (33.3)	16 (76.2)	6 (28.6)	4 (19.0)
			2008–2014	49	19 (38.7)	40 (81.6)	17 (34.7)	7 (14.2)
			2014	45^∗^	19 (42.2)	35 (77.7)	16 (35.6)	7 (15.6)
			2008–2014	94^∗∗^	38 (40.4)	75 (79.8)	33 (35.1)	14 (14.9)
2	Northwest	Saint Petersburg	Before 2001	18	4 (22.2)	15 (83.3)	3 (16.7)	2 (11.1)
3	Southern	Rostov-on-Don	2008	8	0 (0)	8 (100)	0 (0)	0 (0)
4	Ural	Yekaterinburg	2008	3	1 (33.3)	3 (100)	1 (33.3)	0 (0)
5	Siberian	Novosibirsk	Before 2005	29	6 (20.8)	22 (75.9)	6 (20.7)	7 (23.8)
6		Kyzyl	2008	6	1 (16.7)	4 (66.7)	0 (0)	1 (16.7)
7	Far Eastern	Yakutsk	2008	7	1 (14.3)	6 (85.7)	1 (14.3)	1 (14.3)
8		Khabarovsk	2009	24	8 (33.3)	19 (79.2)	8 (33.3)	5 (20.8)

^∗^Intravenous drug users (IDUs); ^∗∗^all samples including IDUs.

## Supplementary Materials

Supplementary 1 Figure S1: geographical origins of samples of hepatitis C patients infected with HCV 1b from the Russian Federation used in the analysis of frequency of substitutions in HCV core amino acid positions 70 and 91. Data on the sample set is presented in Table 2.

Supplementary 2 Figure S2: phylogenetic tree for the complete HCV core sequences covering nucleotide positions 342–915 according to HCV 1a reference strain H77 (GenBank accession number NC004102) built using a Bayesian likelihood-based algorithm; detailed overview of the tree fragments (А; В). Sequences of the Russian origins are underlined. Sequences with wild type aa in positions 70 and 91 (R70, L91) are colored green; with mutated R70Q/H, orange; with M91L substitution, blue; with substitutions in both 70 and 91(R70Q/H, M91L), red.

Supplementary 3 Figure S3: T-cell epitopes in the consensus amino acid sequence of nucleocapsid (core) of HCV 1b encompassing amino acids 70 (A) and 91 (B) localized using Immune Epitope Database and Analysis resource (http://www.iedb.org).

Supplementary 4 Table S1: presentation by HLA I alleles of 9-mer peptide variants derived from the region between aa 62 and 78 of the nucleocapsid (core) of HCV 1b encompassing aa 70, predicted using EPISPOT tool (http://bio.med.ucm.es/episopt.html; EPISPOT [25]). Nine amino acid long peptides were chosen based on the consensus amino acid sequence of nucleocapsid (core) protein of HCV 1b variants isolated in the Russian Federation. Upper row: position of amino acids in HCV core; % (*n* = 189), frequency of occurrence of the respective variant in a set of 189 HCV 1b sequences; potential epitopes, selection of potential CTL-epitopes localized within the peptide seqeunce; PPC, population protection coverage (PPC) calculated as the proportion of the population that can potentially mount an immune response to the peptide computed using known frequences of the HLA I alleles that can present the peptide; HLA class I binding profile lists HLA class I molecules predicted to present the peptide. NF, not found.

Supplementary 5 Table S2: presentation by HLA I alleles of 9-mer peptide variants derived from the region between aa 83 and 99 of the nucleocapsid (core) of HCV 1b encompassing aa 91, predicted using EPISPOT tool (http://bio.med.ucm.es/episopt.html; EPISPOT [45]). Nine amino acid long peptides were chosen based on the consensus amino acid sequence of nucleocapsid (core) protein of HCV 1b variants isolated in the Russian Federation. Upper row: position of amino acids in HCV core; % (*n* = 189), percentage of respective variant in a set of 189 HCV 1b sequences; PPC, population protection coverage (PPC) calculated as the proportion of the population that can potentially mount an immune response to the peptide computed using known frequences of the HLA I alleles that can present the epitope; HLA I binding profile lists HLA I molecules predicted to present the epitope. NF, not found.

Supplementary 6 Table S3: NetMHCIIpan 3.1 engine [48] predicts only weak binding of HCV 1b core peptides encompassing amino acid residue 91 to a limited number of human HLA class II molecules. Peptide predicted to be recognized is boxed in green and aa position 91 in red. Strong binding is characterized by binding level < 2%; weak binding, 2 to 10%; no binding, >10%.
