# Supplementary figures and images for: Frequency of Interferon-Resistance Conferring Substitutions in Amino Acid Positions 70 and 91 of Core Protein of the Russian HCV 1b Isolates Analyzed in the T-Cell Epitopic Context
Source: J Immunol Res. 2018 Feb 7;2018:7685371. doi: 10.1155/2018/7685371 (PMC5821972; doi:10.1155/2018/7685371)

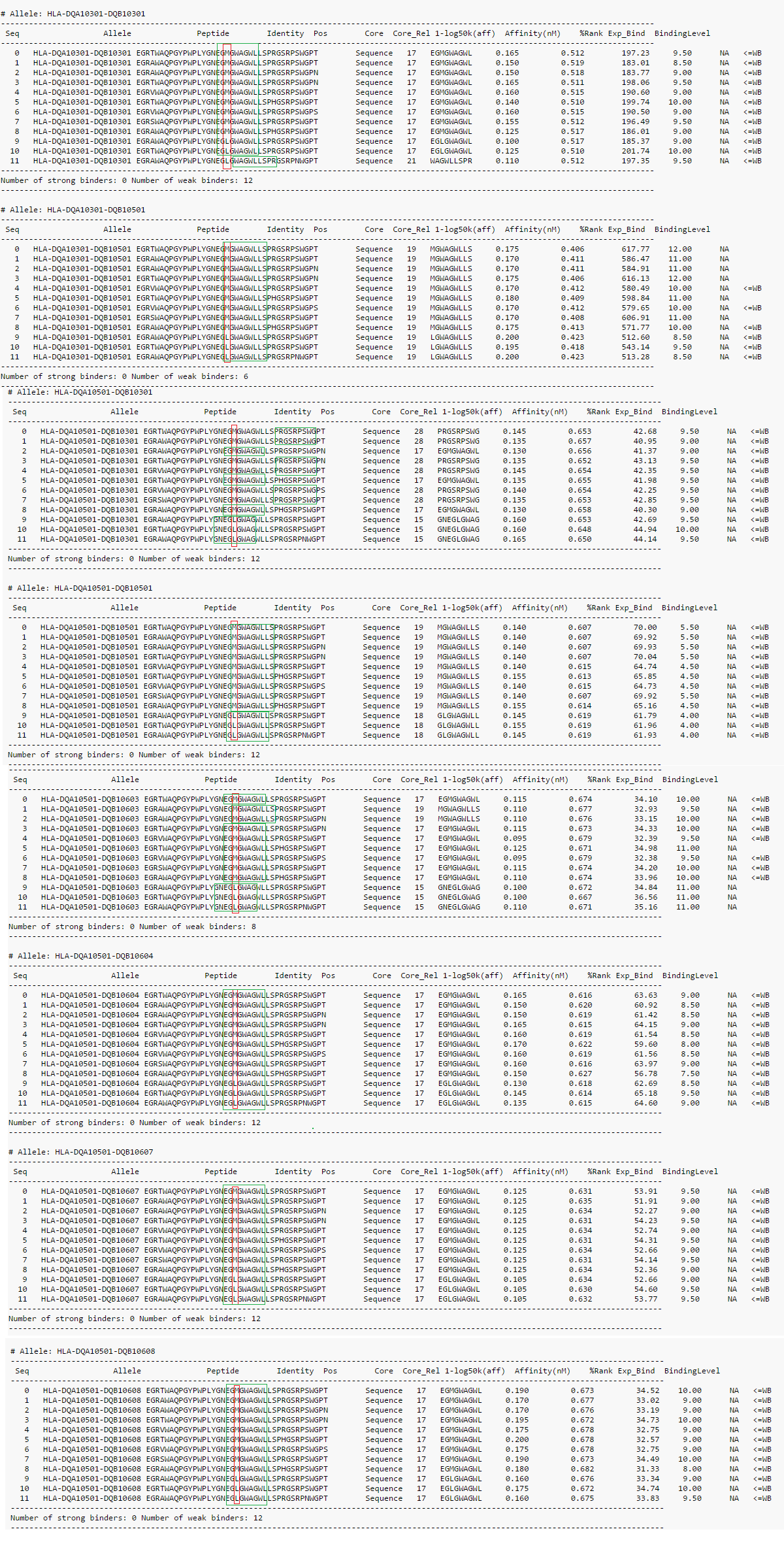

Supplement: Supplementary 6 — Table S3: NetMHCIIpan 3.1 engine [48] predicts only weak binding of HCV 1b core peptides encompassing amino acid residue 91 to a limited number of human HLA class II molecules. Peptide predicted to be recognized is boxed in green and aa position 91 in red. Strong binding is characterized by binding level < 2%; weak binding, 2 to 10%; no binding, >10%. [file 7685371.f6.png]
